# Ductular reaction correlates with fibrogenesis but does not contribute to liver regeneration in experimental fibrosis models

**DOI:** 10.1371/journal.pone.0176518

**Published:** 2017-04-26

**Authors:** András Rókusz, Dániel Veres, Armanda Szücs, Edina Bugyik, Miklós Mózes, Sándor Paku, Péter Nagy, Katalin Dezső

**Affiliations:** 1 First Department of Pathology and Experimental Cancer Research, Semmelweis University, Budapest, Hungary; 2 Department of Biophysics and Radiation Biology, Semmelweis University, Budapest, Hungary; 3 Institute of Pathophysiology, Semmelweis University, Budapest, Hungary; 4 Tumor Progression Research Group, Joint Research Organization of the Hungarian Academy of Sciences and Semmelweis University, Budapest, Hungary; University of Navarra School of Medicine and Center for Applied Medical Research (CIMA), SPAIN

## Abstract

**Background and aims:**

Ductular reaction is a standard component of fibrotic liver tissue but its function is largely unknown. It is supposed to interact with the matrix producing myofibroblasts and compensate the declining regenerative capacity of hepatocytes. The relationship between the extent of fibrosis—ductular reaction, proliferative activity of hepatocytes and ductular reaction were studied sequentially in experimental hepatic fibrosis models.

**Methods:**

Liver fibrosis/cirrhosis was induced in wild type and TGFβ overproducing transgenic mice by carbon tetrachloride and thioacetamide administration. The effect of thioacetamide was modulated by treatment with imatinib and erlotinib. The extent of ductular reaction and fibrosis was measured by morphometry following cytokeratin 19 immunofluorescent labeling and Picro Sirius staining respectively. The proliferative activity of hepatocytes and ductular reaction was evaluated by BrdU incorporation. The temporal distribution of the parameters was followed and compared within and between different experimental groups.

**Results:**

There was a strong significant correlation between the extent of fibrosis and ductular reaction in each experimental group. Although imatinib and erlotinib temporarily decreased fibrosis this effect later disappeared. We could not observe negative correlation between the proliferation of hepatocytes and ductular reaction in any of the investigated models.

**Conclusions:**

The stringent connection between ductular reaction and fibrosis, which cannot be influenced by any of our treatment regimens, suggests that there is a close mutual interaction between them instead of a unidirectional causal relationship. Our results confirm a close connection between DR and fibrogenesis. However, since the two parameters changed together we could not establish a causal relationship and were unable to reveal which was the primary event. The lack of inverse correlation between the proliferation of hepatocytes and ductular reaction questions that ductular reaction can compensate for the failing regenerative activity of hepatocytes. No evidences support the persistent antifibrotic property of imatinib or erlotinib.

## Introduction

Chronic damage of liver tissue causes gradual accumulation of extracellular matrix (ECM), fibrosis, which can eventually progress to complete architectural reconstruction termed cirrhosis. Hepatic fibrosis/cirrhosis causes organ dysfunction and other complications resulting in common clinical problems. Yet, there are numerous unresolved questions regarding the pathogenesis of this severe disease, e.g. the role and function of hepatic progenitor cells in fibrogenesis and regeneration. Small epithelial tubules called “bile duct proliferation” were observed along the fibrotic septa of cirrhotic livers a long time ago but no special attention was paid to them. Now, these tubules are referred to as ductular reaction and they are thought to represent hepatic progenitor cells [1]. Their closed spatial and functional relationship with myofibroblasts put them in the limelight [2–5], since myofibroblasts are the major source of the deposited collagenous matrix. The correlation between the extent of ductular reaction and fibrosis across a range of liver pathologies raises the question if there is a causal relationship between them [6]. Ductular reaction may also play a favourable role in liver cirrhosis. Originally Falkowski et al. [7] proposed that the ductular reaction may be an alternative regenerative pathway, which is activated when the replicative capacity of the senescent hepatocytes is compromised. This view was later supported by the description of hepatocytic differentiation of ductular progenitor cells [8, 9]. This simple and attractive model, however, is mostly based on static tissue analysis and contradictory observations have recently emerged. The progenitor cell origin of regenerating hepatocytes was excluded in several experimental models applying the cre-lox based lineage tracing technique [10–12]. Others and we [13, 14] failed to determine inverse relationship between the proliferative activity of hepatocytes and ductular reaction in human cirrhotic livers. These considerations led us to perform experiments where the dynamics of fibrosis, ductular reaction and hepatocyte proliferation can be continually monitored throughout the development of cirrhosis. Furthermore, our aim was to examine if the potentially favourable and unfavourable consequences of ductular reaction can be separated.

Liver fibrosis was induced in wild type C57Bl/6 mice by chronic administration of thioacetamide (TA) and carbon tetrachloride/phenobarbital (CCl_4_/PhB). The TA experiments were also performed on transgenic mice overexpressing active transforming growth factor beta 1 (TGFβ1) in the liver [15]. TGFβ is probably the most pleiotropic growth factor with major influence on hepatocyte and ductular proliferation, as well as on liver fibrosis [16].

The TA administration was also combined with two drugs. Imatinib and erlotinib are widely used tyrosine kinase inhibitors. The primary target of erlotinib is epidermal growth factor receptor (EGFR) while imatinib has a broader spectrum. It was originally designed for the treatment of chronic myelogenous leukaemia by blocking the activity of bcr/abl tyrosine kinase but turned out to be an efficient inhibitor of c-kit and platelet-derived growth factor receptor (PDGFR) as well [17]. Both compounds are used for the treatment of different malignant tumors and imatinib has been successfully applied in human patients as an antifibrotic agent [18]. They have also shown antifibrotic activity in several experimental hepatic fibrosis models [19–22], but little is known about their effect on the behavior of ductular reaction and hepatocytes.

All fibrogenic protocols induced progressive hepatic fibrosis. There was a strong positive correlation between the extent of ductular reaction and fibrosis in each model. However, the compensatory growth function of ductular reaction could not be observed. Both imatinib and erlotinib had temporary antifibrotic effect but eventually, and under adverse conditions (TGFβ overexpression), they were inefficient. Neither drug had antifibrotic effect on established cirrhosis induced by TA treatment.

## Materials and methods

### Animal experiments

**Fig 1:**
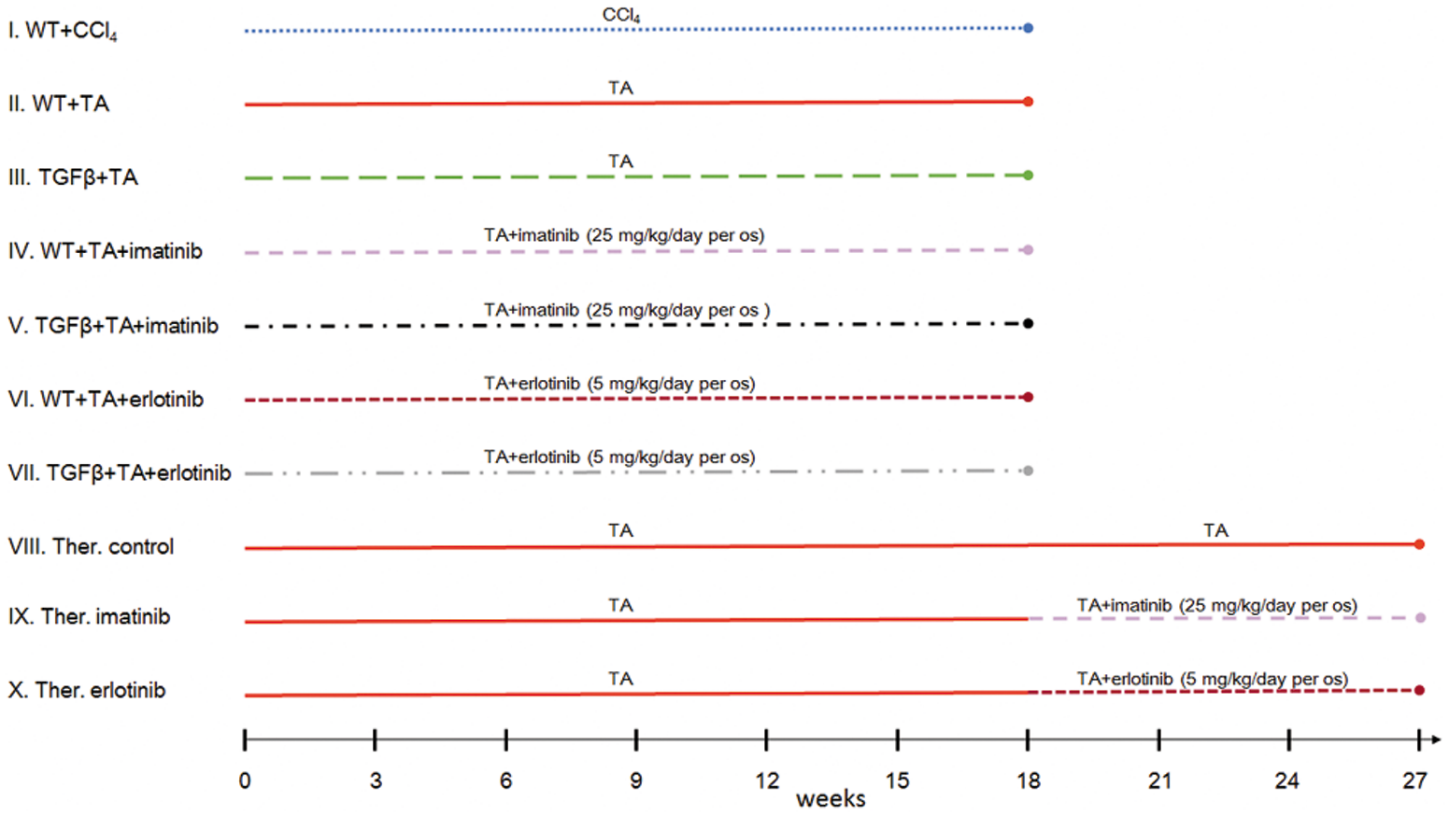
Schematic representation of the different experimental groups.

All experiments were conducted on 8 weeks old male C57Bl/6 mice inbred in our Institute. All animals were housed under controlled temperature with a 12 hour light-dark cycle, and had access to drinking water ad libitum. Ten experimental groups were formed (Fig 1):

Basic models:

I Hepatic fibrosis was induced by carbon tetrachloride/phenobarbital treatment (CCl_4_ 0,2ml/kg, dissolved in sunflower oil, twice a week per os/PhB 0,5g/l in drinking water) in wild type mice (WT+CCl_4_; n = 49),
II or by TA administration (300 mg/l in drinking water) in wild type (WT+TA; n = 50) and
III transgenic mice overexpressing active TGFβ in hepatocytes [15] (TGFβ+TA; n = 61) (the mice were a kind gift from Snorri S. Thorgeirsson).

Drug treated models:

IV Imatinib (Glivec, Novartis, Basel; 25 mg/kg, dissolved in water, per os) was given daily to TA treated wild type mice (WT+TA+imatinib; n = 54) or
V TGFβ transgenic mice (TGFβ+TA+imatinib; n = 38).
VI Erlotinib (Tarceva, Roche, Basel; 5 mg/kg, dissolved in dimethyl sulfoxide (DMSO) then water) was given daily, beside TA treatment, to both wild type (WT+TA+erlotinib; n = 48) and
VII TGFβ transgenic mice (TGFβ+TA+erlotinib; n = 38).

The animals were sacrificed after 3, 6, 9, 12, 15 and 18 weeks of treatment. Each time point represents 5–16 animals.

Therapeutic models:

VIII TA (300 mg/l in drinking water) was given to wild type mice for a maximum of 27 weeks (Ther. control; n = 18).
IX Imatinib treatment (as described above) was started on the 19th week in addition to TA (Ther. imatinib; n = 18).
X Erlotinib treatment was started on the 19th week (as described above) in addition to TA (Ther. erlotinib; n = 17).

Animals were sacrificed at 3, 6 and 9 weeks after the initiation of pharmacological treatment (in other words on the 21^st^, 24^th^ and 27^th^ week of the experiment). Each time point represents 5–7 animals.

Each animal received three doses of bromodeoxyuridine (BrdU; 500 mg/kg) intraperitoneally, 20, 2 and 1 hour before termination. After humanely sacrificing the animals using cervical dislocation, samples from the liver were fixed for histological analysis, the rest was snap-frozen in liquid nitrogen. The animal study protocols were conducted according to National Institute of Health (NIH) guidelines for animal care and were approved by the Institutional Animal Care and Use Committee of Semmelweis University (Permit Number: PEI/001/1730-12/2015).

### Morphometric analysis

From each animal four parameters were measured/counted: the extent of fibrosis and ductular reaction, as well as the proliferative activity of hepatocytes and ductular reaction.

For the morphometric analysis of fibrosis three images from Picro Sirius stained sections were captured with a Zeiss Axioskop 2 plus microscope (Zeiss, Oberkochen, Germany) using a 5x objective and evaluated by the Quick PhotoMicro 2.2 software (Promicra, Prague, Czech Republic).

The area occupied by ductular reaction was measured on cytokeratin 19 (CK19) immunostained frozen sections (rat monoclonal anti-CK 19 antibody; cat. no. TROMA-III; Developmental Studies Hybridoma Bank, Iowa City, IA; dil.:1:200). From each liver three images were captured with a Bio-Rad confocal system (MRC 1024; Bio-Rad, Richmond, CA) and evaluated by the ImageJ 1.49k program (NIH, Bethesda, MD).

The incorporated BrdU was immunostained (mouse monoclonal anti-BrdU antibody; cat. no.: 347580; BD Biosciences, Franklin Lakes, NJ; dil.: 1:20) as described before [23]. 5000 hepatocytes and 500 ductular cells were counted, the percentage of BrdU-positive cells was given as a result.

### Statistical analysis

Statistical analysis was performed with StatSoft Statistica software (StatSoft Inc., Tulsa, OK; version 8.0). The deviation from Gaussian distribution of variables was tested with Kolmogorov-Smirnov and Lilliefors’ method. The normality condition was fulfilled for all the 4 variables in each group, therefore 2-way factorial ANOVA was performed with a Tukey-Kramer HSD test (with unequal sample size). Correlation between variables was tested using Spearman rank correlation test (a few equal values were in the dataset) and Spearman correlation coefficients were determined. Results were considered significant at a p value less than or equal to 0,05. For characterising the differences between groups both means and medians were calculated and data were visualized on scatter and box plots.

## Results

### Basic models

**Fig 2:**
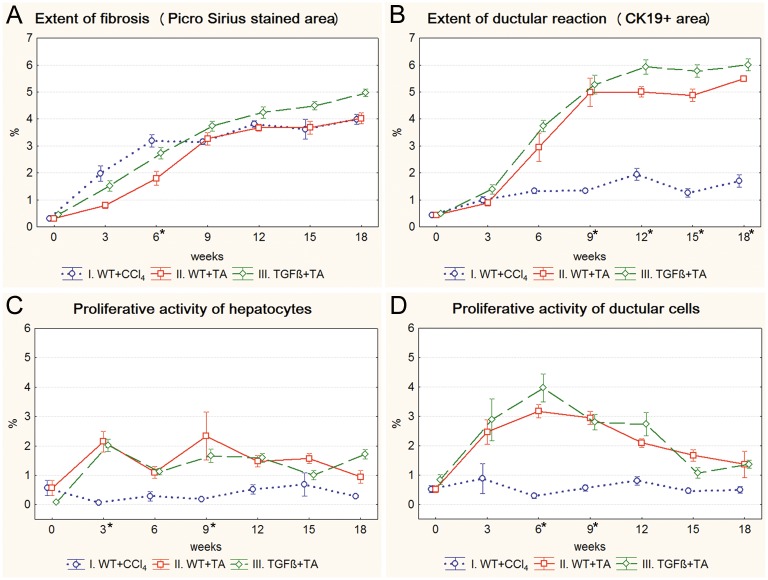
Results of the basic models (I. WT+CCl_4_, II. WT+TA, III. TGFβ+TA). Data are represented as means ± standard error of the mean (SEM). * marks time points, where there was a significant difference between the results of the WT+CCl_4_ and WT+TA groups. The extent of fibrosis is significantly higher in the WT+CCl_4_ group on the 6^th^ week of treatment (**A**), while the extent of ductular recation is significantly lower from the 9^th^ week until the end of the experiment (**B**). The proliferative activity of hepatocytes (**C**) and ductular reaction (**D**) is significantly lower on the 3^rd^ and 9^th^, or on the 6^th^ and 9th week of the experiment, respectively.

The fibrosis was progressive in each model and completely circumscribed pseudolobules were formed by the end of the experiment (S1 Fig). The advancement was faster at the beginning and then slowed down, especially in the CCl_4_ treated mice (Fig 2A). As it was expected, the fibrosis was more severe at each time point in the TGFβ+TA group compared to wild type mice (WT+TA group) (Fig 2A). The extent of ductular reaction, quantitated by the measurement of CK19-positive area, was in line with the Picro Sirius data in the TA models (Fig 2A and 2B and S2 Fig). It showed a slightly increasing trend in the CCl_4_ treated mice as well but from the 9^th^ week it was significantly lower compared to the TA groups. Interestingly, the extent of ductular reaction was similar in the CCl_4_ and TA treated wild type mice on the 3^rd^ week, despite the difference in the amount of fibrosis. There was a divergence between the TA and CCl_4_ treated animals in the cell proliferation data as well. In the TA groups, a temporary rise in both the proliferation of hepatocytes and ductular reaction was followed by a gradual decline. CCl_4_ induced much lower levels of cell proliferation with a relatively stable trend in both cell types (Fig 2C and 2D).

### Drug treated models

**Fig 3:**
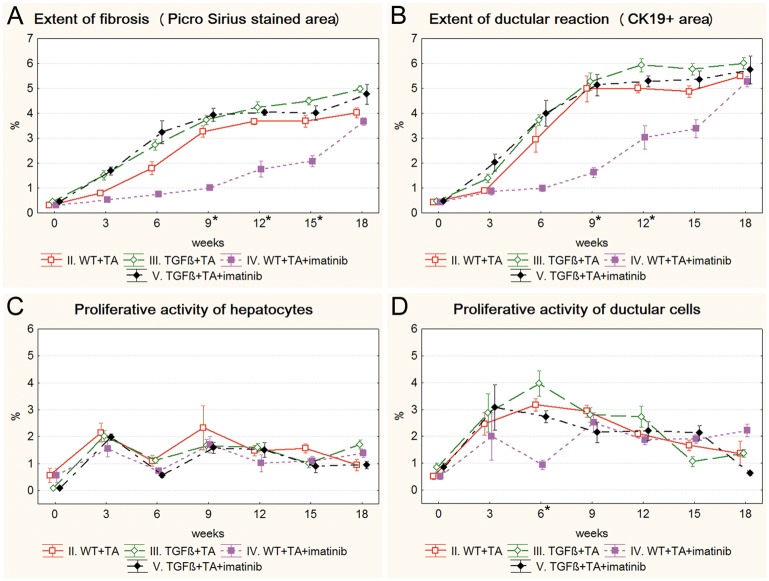
Results of the imatinib treated groups (IV. WT+TA+imatinib, V. TGFβ+TA+imatinib) and their control groups (II. WT+TA, III. TGFβ+TA). Data are represented as means ± standard error of the mean (SEM).* marks time points, where there was a significant difference between the results of the WT+TA and WT+TA+imatinib groups. Imatinib treatment temporarily resulted in significantly lower extent of fibrosis (**A**, 9^th^, 12^th^, 15^th^ week) and ductular reaction (**B**, 9^th^ and 12^th^ week) in wild type mice. The proliferative activity of ductular reaction was also significantly lower on the 6^th^ week of the experiment in imatinib treated wild type mice (**D**). Imatinib treatment did not have any significant effect on TGFβ transgenic mice.

**Fig 4:**
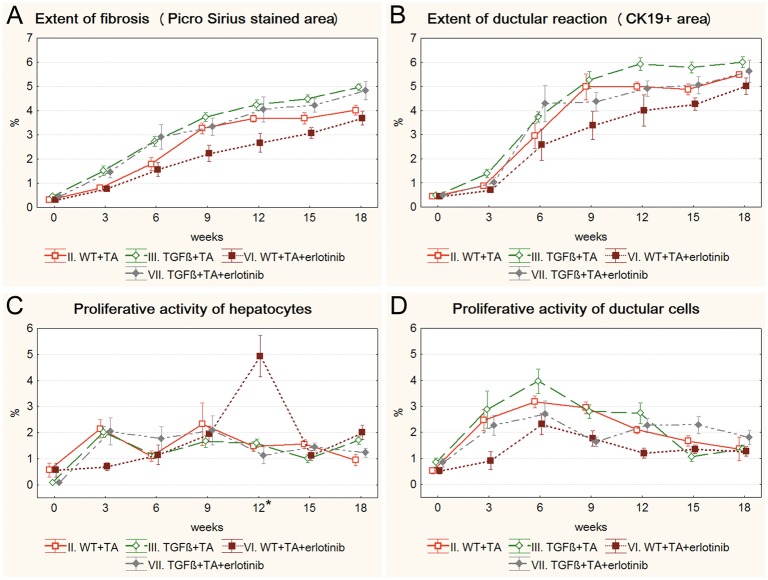
Results of the erlotinib treated groups (VI. WT+TA+erlotinib, VII. TGFβ+TA+erlotinib) and their control groups (II. WT+TA, III. TGFβ+TA). Data are represented as means ± standard error of the mean (SEM). * marks the time point, where there was a significant difference between the results of the WT+TA and WT+TA+erlotinib groups. Erlotinib treatment resulted in significantly higher hepatocyte proliferation on the 12^th^ week of the experiment (**C**). Erlotinib treatment did not have any significant effect on TGFβ transgenic mice.

Both imatinib and erlotinib suppressed the progression of fibrosis and ductular reaction temporarily in wild type mice (Figs 3A, 3B, 4A and 4B). Imatinib treatment resulted in a significant decrease in the extent of fibrosis on the 9^th^, 12^th^ and 15^th^ week (Fig 3A), and in the extent of ductular reaction on the 9^th^ and 12^th^ week of the experiment (Fig 3B) (S3 and S4 Figs). The effect of erlotinib did not reach significant levels in any of the time points (Fig 4A and 4B). However, at the end of the experiment, due to a compensatory growth, there was no difference in the degree of fibrosis or ductular reaction between the three groups of TA treated wild type mice. Not even a temporary inhibition as described above could be observed in the TGFβ transgenic mice (S5 and S6 Figs). None of the applied drugs had a lasting influence on the proliferative activity of hepatocytes or ductular reaction (Figs 3C, 3D, 4C and 4D).

### Therapeutic models

**Fig 5:**
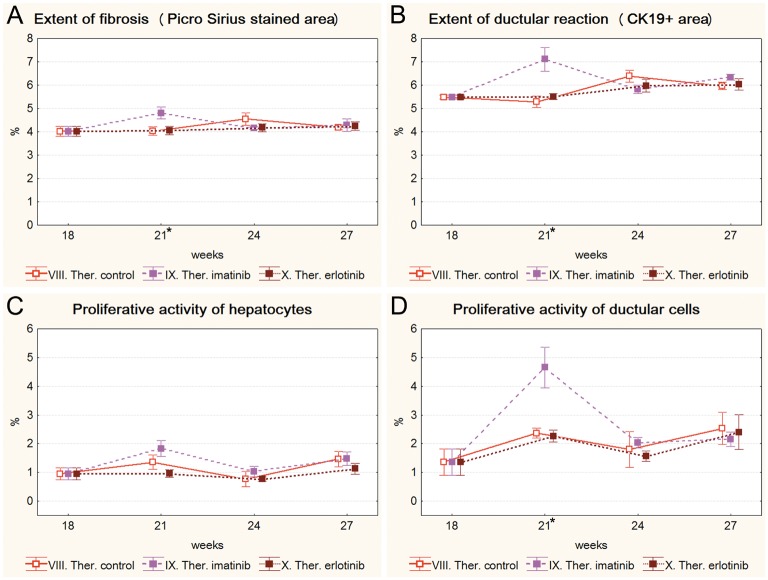
Results of the therapeutic experiment (VIII. Ther. control, IX. Ther. imatinib, X. Ther. erlotinib). Data are represented as means ± standard error of the mean (SEM). * marks the time points, where there was a significant difference between the results of the Ther. imatinib and Ther. control groups. Imatinib treatment resulted in significantly higher extent of fibrosis (**A**); extent (**B**), and proliferative activity (**D**) of ductular reaction on the 21^st^ week of the experiment (after 3 weeks of imatinib treatment). Erlotinib treatment did not have any significant effect on livers with established fibrosis.

Both imatinib and erlotinib were used in a “therapeutic” experiment to simulate a clinical situation and test if these drugs can influence preexistent fibrosis. The administration of both tyrosine kinase inhibitors was started after 18 weeks of TA treatment and was continued further on (S7 and S8 Figs). Neither of the drugs were able to block the progression of fibrosis (Fig 5A). Interestingly, imatinib even induced a significant temporary increase in the extent of Picro Sirius staining, and the extent and proliferative activity of ductular reaction (Fig 5A, 5B and 5D).

### Correlation analysis

To investigate the relationship between the studied parameters without considering the timewise distribution of the data, a correlation analysis was performed between each pair of parameters in the different experimental groups. The results are shown in Table 1. The extent of fibrosis showed a strong positive correlation with the extent of ductular reaction in each model (S9 Fig). This correlation was the weakest in the CCl_4_ group. Surprisingly, the proliferative activity of ductular reaction correlated negatively in three groups with Picro Sirius (S10A, S10B and S10D Fig) and in one group with CK19 staining (S11A Fig) indicating an inverse relationship between the extent of fibrosis/ductular reaction and the proliferative activity of ductular reaction. Contrary to our expectations, the proliferation of hepatocytes correlated positively in two groups with Picro Sirius (S10E and S10F Fig) and CK19 (S11C and S11D Fig) staining or the proliferative activity of ductular reaction (S11E and S11F Fig). Furthermore, no negative correlation was found in any of the groups between the proliferation of hepatocytes and the extent or proliferation of the ductular reaction.

**Table 1:** Correlation between the analysed parameters.

	Picro-CK19	*p*	Picro-hep	*p*	Picro-duct	*p*	CK19-hep	*p*	CK19-duct	*p*	hep- duct	*p*
I. WT+CCl_4_ (n = 49)	**0.66**	*<0*.*001*	**0.48**	*<0*.*001*	0.22	*ns*	**0.37**	*0*.*009*	0.24	*ns*	0.23	*ns*
II. WT+TA (n = 50)	**0.83**	*<0*.*001*	0.13	*ns*	**-0.33**	*0*.*018*	0.18	*ns*	-0.12	*ns*	0.02	*ns*
III. TGFβ+TA (n = 61)	**0.81**	*<0*.*001*	0.03	*ns*	**-0.56**	*<0*.*001*	0.01	*ns*	**-0.41**	*<0*.*001*	0.09	*ns*
IV. WT+TA+imatinib (n = 54)	**0.94**	*<0*.*001*	0.19	*ns*	**0.31**	*0*.*025*	0.17	*ns*	**0.33**	*0*.*015*	**0.32**	*0*.*002*
V. TGFβ+TA+imatinib (n = 38)	**0.81**	*<0*.*001*	-0.11	*ns*	**-0.46**	*0*.*004*	-0.09	*ns*	-0.32	*ns*	0.12	*ns*
VI. WT+TA+erlotinib (n = 48)	**0.92**	*<0*.*001*	**0.53**	*<0*.*001*	0.03	*ns*	**0.55**	*<0*.*001*	0.13	*ns*	0.09	*ns*
VII. TGFβ+TA+erlotinib (n = 38)	**0.89**	*<0*.*001*	-0.01	*ns*	-0.2	*ns*	-0.01	*ns*	-0.28	*ns*	-0.29	*ns*
VIII. Ther. control (n = 18)	**0.61**	*<0*.*001*	-0.33	*ns*	0.03	*ns*	-0.34	*ns*	-0.37	*ns*	0.3	*ns*
IX. Ther. imatinib (n = 18)	**0.63**	*<0*.*001*	0.2	*ns*	0.37	*ns*	0.4	*ns*	0.26	*ns*	**0.47**	*0*.*048*
X. Ther. erlotinib (n = 17)	**0.81**	*<0*.*001*	0.02	*ns*	0.09	*ns*	-0.02	*ns*	-0.13	*ns*	0.33	*ns*

The values represent Spearman’s correlation coefficients (r_s_). ns—not significant; Picro—extent of fibrosis; CK19 –extent of ductular reaction; hep—proliferative activity of hepatocytes, duct—proliferative activity of ductular reaction.

## Discussion

Liver fibrosis/cirrhosis was induced in mice by the two widely used fibrogenic agents, TA and CCl_4_. The TA model was diversified to modulate the histological reaction. Each experimental groups reached the cirrhotic stage. The primary purpose of the experiment was to analyse the dynamics and relationship of three important components of the fibrotic process: (i) the extent of fibrosis, (ii) the proliferation of hepatocytes and (iii) the extent and proliferative activity of ductular reaction. Morphometric analysis of the extent of fibrosis is more reliable for the evaluation of fibrosis progression than the use of staging systems [24]; hence we quantitated the Picro Sirius stained slides. The extent of the ductular reaction was followed by CK19 immunostaining. Cell proliferation was examined by the immunohistochemical detection of BrdU-positive cells.

The results were evaluated in two different ways. The dynamics of timewise changes in the various parameters were visualized on 2D graphs, while the connection between any two parameters within each group was studied by correlation analysis regardless of the time point when the data were collected. This latter kind of analysis is similar to human investigations, when only the actual parameters can be examined.

The basic concept to be addressed is that, with the advancement of fibrosis, the regenerative activity of the hepatocytes declines but it is compensated by the contribution of ductular reaction.

In each experimental model, there is a strong correlation between the extent of fibrosis and ductular reaction. This observation is in line with previous results of a wide variety of human studies [2, 25–27]. Surprisingly, CCl_4_ induces a much milder ductular reaction than TA but the extent of fibrosis is comparable in the two models. That is, one “unit” of ductular reaction in the CCl_4_ experiment is associated with a larger amount of fibrosis. Therefore, although the extent of fibrosis and ductular reaction correlate, their ratio depends on the initiating event. Interestingly, we could not detect correlation between the extent of ductular reaction and etiology in human cirrhotic livers but the fibrotic area was larger in non-viral hepatitis than in viral hepatitis related cases [14], which also indicates that the ratio of ductular reaction and fibrosis varies according to the etiology. Ductular reaction can potentially contribute to fibrogenesis by transition into myofibroblasts (epithelial-to-mesenchymal transition—EMT) [28] but this aspect has not been addressed in the present study.

Another important and unresolved issue is which event is the primary one and which one is the consequence? Van Hul et al. [3] described that the increased expression of matrix components preceded the elevation of ductular markers with 4–7 days. Human observations in NASH [26], HCV infection related fibrosis [2, 29] and in vivo experimental models [30, 31] suggest that ductular reaction drives fibrosis, while others [3, 32] propose that ECM deposition or remodeling is required for the expansion of ductular reaction. The dynamics of these two parameters were very similar in all of our experimental groups, although we have to admit that our 3 week observation intervals might have been too long to detect a brief shift in the emergence of fibrosis and ductular reaction (such experiments with shorter intervals are in progress). The modifications of TA treatment can be regarded as a functional approach for this problem. Erlotinib most likely influences the fibrotic process through the inhibition of ductular reaction [33] while TGFβ and imatinib affect myofibroblasts (in the opposite direction). Neither of these changes resulted in separation of the progression of fibrosis and ductular reaction. Our results and most of the cited data are consistent with the proposal of Desmet [34] suggesting that the formation of ductular reaction in cirrhosis depends on the mutual interaction of the emerging ductular structures and myofibroblasts regulated by several feedback mechanisms and therefore this “chicken or egg” paradigm [6] cannot be resolved.

Regeneration is another important aspect of liver cirrhosis. Hepatocyte proliferation increases sharply upon the start of TA treatment then declines gradually in all models. Surprisingly, the trend of the proliferative activity of ductular reaction is similar, i.e. an early surge is followed by decline. The correlation analysis does not indicate increased activity of ductular proliferation in fibrotic livers either. In fact, in 3 out of the 6 TA models there is a significant negative correlation between the proliferation of ductular reaction and the extent of fibrosis. The proliferative activity of both hepatocytes and ductular reaction remains on a continuously low level in the CCl_4_ treated mice. Therefore, although the extent of ductular reaction increases with fibrosis, the activity of ductular proliferation does not. No negative correlation could be detected between the proliferation of hepatocytes and ductular reaction in any of the models. Therefore, our data do not support the existence of an inverse linkage between declining hepatocyte and increasing ductular proliferation during the progression of liver fibrosis. The Ki-67 index of hepatocytes and ductular reaction showed a positive correlation in advanced human cirrhotic cases [14] and Eleazar et al. [13] did not find any inverse correlation between the proliferation of hepatocytes and ductular reaction in chronic hepatitis either. In our models, the expanding ductular reaction together with a declining proliferative activity do not support the regenerative role of ductular reaction during fibrosis progression. If a large number of ductular cells regenerated the liver parenchyma, the combination of hepatocytic differentiation and decreasing ductular cell proliferation would result in shrinking ductular reaction. Lin et al. [35] provided convincing evidences that in human livers the cirrhotic nodules can be clonal progenies of the ductular reaction. Stueck and Wanless [36] characterised the “budding” of hepatocyte clusters from ductular reaction on the sites of parenchymal extinction in detail. While such “focal” differentiation event cannot be ruled out by our results, our data challenge a steady state flux of ductular reaction into hepatocytes. It should be noted that we did not observe parenchymal extinction in any of the examined livers, hence it is likely that advanced stage of cirrhosis was not reached in our experiment.

Imatinib is thought to reduce fibrosis by blocking the signaling of PDGFR and c-kit. It proved to be efficient in a short term model of TA-induced fibrosis in rat [21]. Imatinib reduced early fibrogenesis in bile duct ligated rats but did not prevent progression when applied in an intervention experiment [19]. It attenuated progenitor cell expansion and inhibited liver tumor formation in choline-deficient, ethionine-supplemented diet (CDE) fed mice [20, 37]. Our recent observations correspond to these results. Imatinib significantly suppressed fibrosis and ductular reaction in the early but not in later time points in wild type mice, and even this transient inhibition was not present in TGFβ transgenic mice. Borkham-Kamphorst et al. [38] reported temporarily increased PDGF/PDGFR expression in early timepoints of an experimental liver fibrosis model, which was followed by sharp downregulation. Such dynamics of PDGF and PDGFR expression could explain the temporary effects of imatinib in our experiment. TGFβ has been also reported to play an important role in acquired imatinib resistance [39–41], this could also explain the complete inefficiency of imatinib in the transgenic mice. Imatinib was also inefficient in the therapeutic experiment.

EGFR activity has been reported in ductular reactions in humans and in experimental animal models [33, 42, 43]. Gene expression analysis indicated that EGFR signaling is associated with the progression of liver fibrosis [44, 45]. These observations gave us the rationale to investigate the impact of erlotinib on liver fibrosis. Erlotinib treatment temporarily suppressed most of the investigated parameters on wild type mice but there was no difference between the control and the treated animals at the endpoint and similar to imatinib it was completely inefficient in the transgenic mice overexpressing TGFβ and in the therapeutic experiment Erlotinib successfully blocked the proliferation of ductular reaction in Nf2-/- mice [46], it also attenuated liver fibrosis in bile duct ligated rats and CCl_4_ treated mice [22]. Inhibition of EGFR signaling delayed but did not block liver regeneration in different experimental models [47, 48] and EGFR inhibitors showed lack of clinical efficacy in human hepatocellular carcinomas [49]. These failures are explained by the redundant growth regulation of these processes. Furthermore, some ligands of EGFR seem to transduce fibrotic, as well as antifibrotic signals [50]. Erlotinib has not shown robust lasting antifibrotic effect in our experimental models. Antagonism between EGFR signaling and TGFβ has also been reported [48]. The complete inefficiency of erlotinib in the TGFβ transgenic mice is in line with this report.

TGFβ is thought to be one of the most important cytokines driving liver fibrosis. It is also one of the most potent inhibitors of hepatocyte proliferation [51]. Since the ductular cells are more resistant to its mitoinhibition, TGFβ has been suggested to be responsible for the compensatory growth of ductules [52]. Our experiment was relevant for all these issues. The increased TGFβ production in the transgenic mice enhanced the extent of fibrosis as described before [53]. However, the inverse proliferative activity of the ductular reaction and hepatocytes could not be observed in mice even with elevated TGFβ production. The hepatocyte proliferation was almost equal in transgenic and wild type mice and the TGFβ+TA was the only experimental group with significant negative correlation between the proliferation of ductular reaction and CK19 staining. That is, the proliferation of ductules decreased with the advancement of ductular reaction. Elevated expression of TGFβ was described in human and experimental cirrhosis as well [16, 54] and it could contribute to the failure of imatinib and erlotinib in our therapeutic experiment. This is a warning if these compounds could be successfully applied for the treatment of liver fibrosis. Further mechanistic experiments would be important to reveal their interaction.

In conclusion, dynamic analysis of the investigated components of hepatic fibrosis confirmed the close relationship between ductular reaction and fibrosis. The strong correlation in several experimental models suggests that these might be two mutually interdependent histological reactions but the ratio of the two components depends on the fibrogenic agent. We could not confirm the inverse relationship between the regenerative activity of hepatocytes and ductular reaction. This failure suggests that the role or function of ductular reaction in fibrotic livers could be substantially different from the traditional “oval cells” in regenerative rodent models [55]. Finally, our results show that neither erlotinib nor imatinib are powerful antifibrotic drugs in these experimental models. The increased TGFβ production during the fibrotic process might contribute to the resistance.

## Supporting information

S1 Fig Progression of fibrosis in the three basic models (A-C: I. WT+CCl_4_; D.-F: II. WT+TA; G-I: III. TGFβ+TA). Representative images from sections with Picro Sirius staining. Scale bar for S1 Fig.: 200μm.
(TIF)

S2 Fig Progression of ductular reaction in the three basic models (A-C: I. WT+CCl_4_; D.-F: II. WT+TA; G-I: III. TGFβ+TA). Representative images from sections with CK19 immunofluorescent labeling. Scale bar for S2 Fig.: 200μm.
(TIF)

S3 Fig Progression of fibrosis in the imatinib treated wild type mice (IV. WT+TA+imatinib; D-F) and their control group (II. WT+TA; A-C). Representative images from Picro Sirius stained sections. Scale bar for S3 Fig.: 200μm.
(TIF)

S4 Fig Progression of ductular reaction in the imatinib treated wild type mice (IV. WT+TA+imatinib; D-F) and their control group (II. WT+TA; A-C). Representative images from sections with CK19 immunofluorescent labeling. Scale bar for S4 Fig.: 200μm.
(TIF)

S5 Fig Progression of fibrosis in the imatinib treated TGFβ transgenic mice (V. TGFβ+TA+imatinib; D-F) and their control group (III. TGFβ+TA; A-C). Representative images from Picro Sirius stained sections. Scale bar for S5 Fig.: 200μm.
(TIF)

S6 Fig Progression of ductular reaction in the imatinib treated TGFβ transgenic mice (V. TGFβ+TA+imatinib; D-F) and their control group (III. TGFβ+TA; A-C). Representative images from sections with CK19 immunofluorescent labeling. Scale bar for S6 Fig.: 200μm.
(TIF)

S7 Fig Progression of fibrosis in the therapeutic models (A-C: VIII. Ther. control; D.-F: IX. Ther. imatinib; G-I: X. Ther. erlotinib). Representative images from sections with Picro Sirius staining. Scale bar for S7 Fig.: 200μm.
(TIF)

S8 Fig Progression of ductular reaction in the therapeutic models (A-C: VIII. Ther. control; D.-F: IX. Ther. imatinib; G-I: X. Ther. erlotinib). Representative images from sections with CK19 immunofluorescent labeling. Scale bar for S8 Fig.: 200μm.
(TIF)

S9 Fig Significant correlations between the extent of fibrosis (Picro Sirius) and the extent of ductular reaction (CK19) in the different experimental groups. Spearman’s correlation coefficients are shown in Table 1.
(TIF)

S10 Fig Significant correlations between the extent of fibrosis (Picro Sirius) and the proliferative activity of ductular reaction (duct. prol.) (A-D); or between the extent of fibrosis (Picro Sirius) and the proliferative activity of hepatocytes (hep. prol.) (E and F) in different experimental groups. Spearman’s correlation coefficients are shown in Table 1.
(TIF)

S11 Fig Significant correlations between the extent of ductular reaction (CK19) and the proliferative activity of ductular reaction (duct. prol.) (A and B); or between the extent of ductular reaction (CK19) and the proliferative activity of hepatocytes (hep. prol.) (C and D); or between the proliferative activity of hepatocytes (hep. prol.) and ductular reaction (duct. prol.) (E and F) in different experimental groups. Spearman’s correlation coefficients are shown in Table 1.
(TIF)

## Abbreviations

BrdU: bromodeoxyuridine
CCl_4_: carbon tetrachloride
CDE: choline-deficient, ethionine-supplemented diet
DEN: diethylnitrosamine
DMSO: dimethyl sulfoxide
ECM: extracellular matrix
EGFR: epidermal growth factor receptor
EMT: epithelial-to-mesenchymal transition
PDGFR: platelet-derived growth factor
PhB: phenobarbital
TA: thioacetamide
TGFβ: transforming growth factor beta
