# Supplementary figures and images for: Ductular reaction correlates with fibrogenesis but does not contribute to liver regeneration in experimental fibrosis models
Source: PLoS One. 2017 Apr 26;12(4):e0176518. doi: 10.1371/journal.pone.0176518 (PMC5405957; doi:10.1371/journal.pone.0176518)

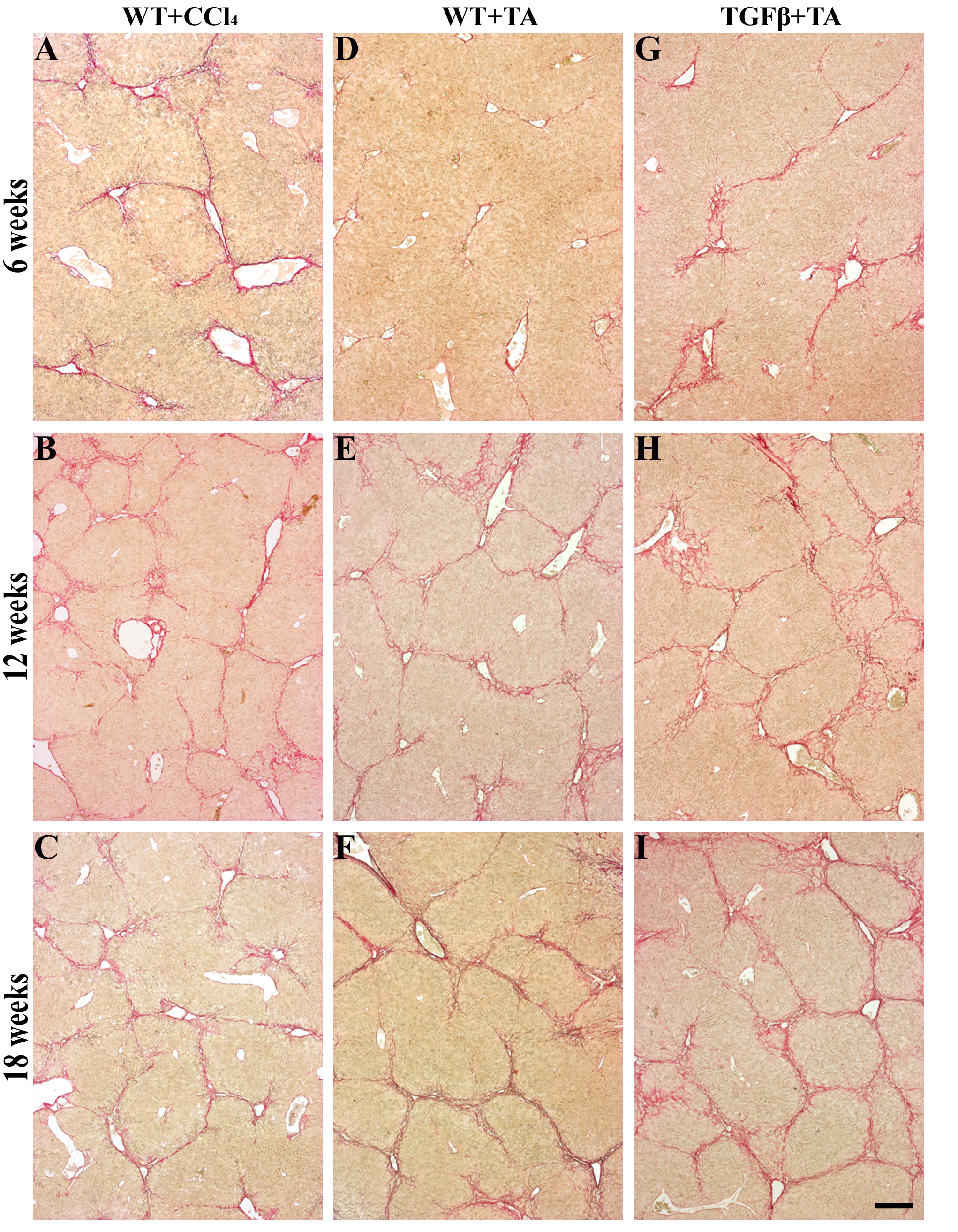

Supplement: S1 Fig — Representative images from sections with Picro Sirius staining. Scale bar for S1 Fig.: 200μm. (TIF) [file pone.0176518.s001.tif]

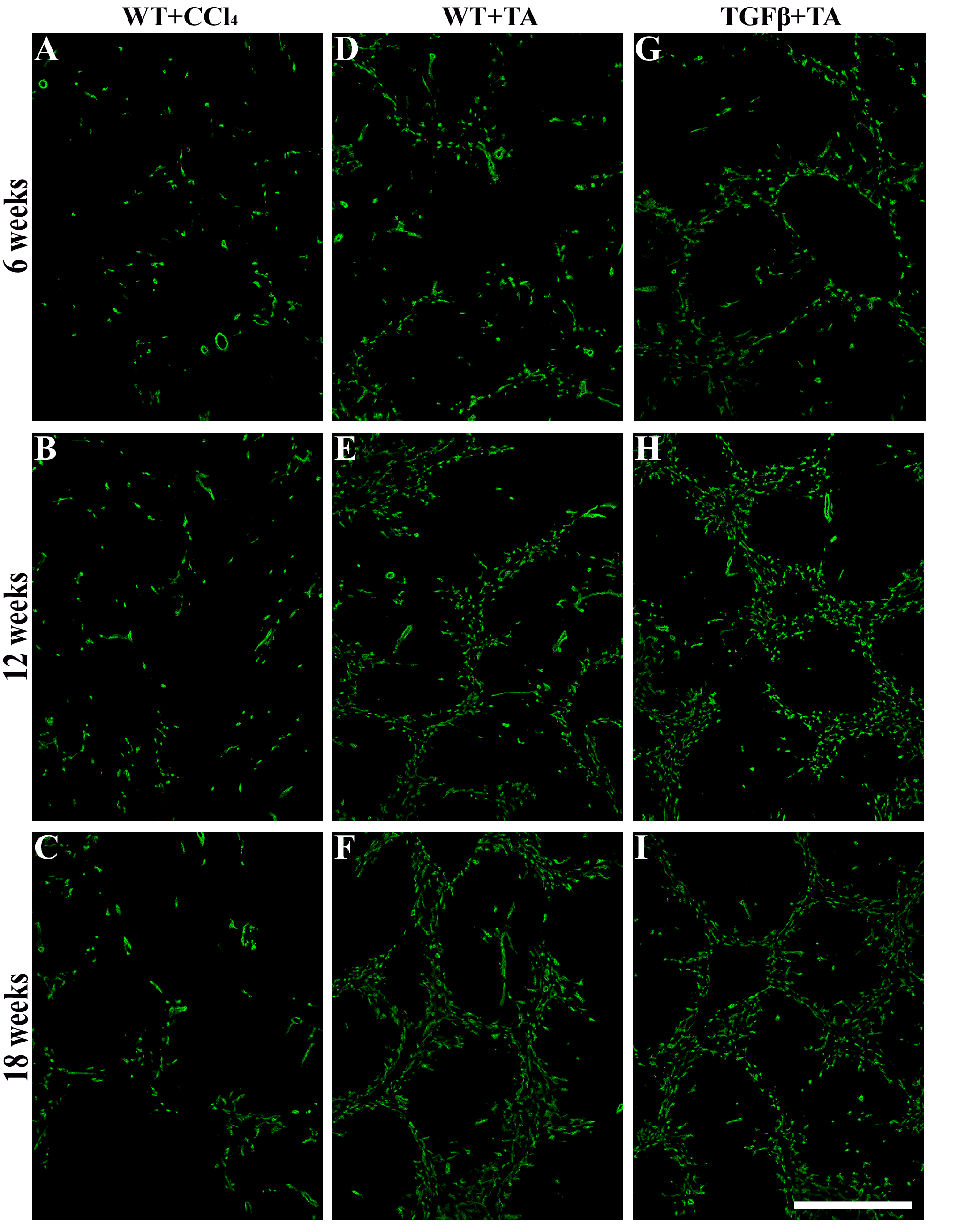

Supplement: S2 Fig — Representative images from sections with CK19 immunofluorescent labeling. Scale bar for S2 Fig.: 200μm. (TIF) [file pone.0176518.s002.tif]

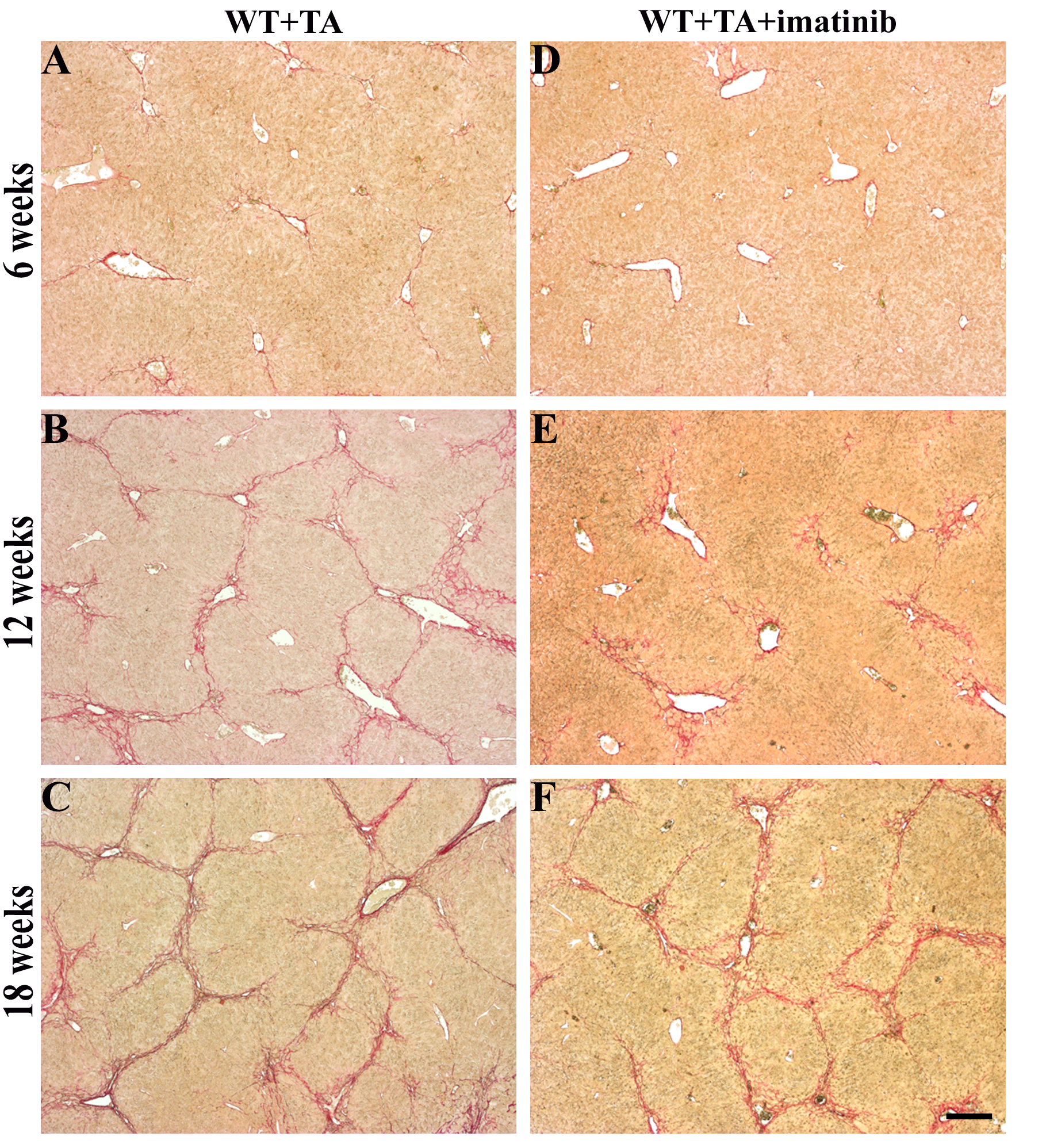

Supplement: S3 Fig — Representative images from Picro Sirius stained sections. Scale bar for S3 Fig.: 200μm. (TIF) [file pone.0176518.s003.tif]

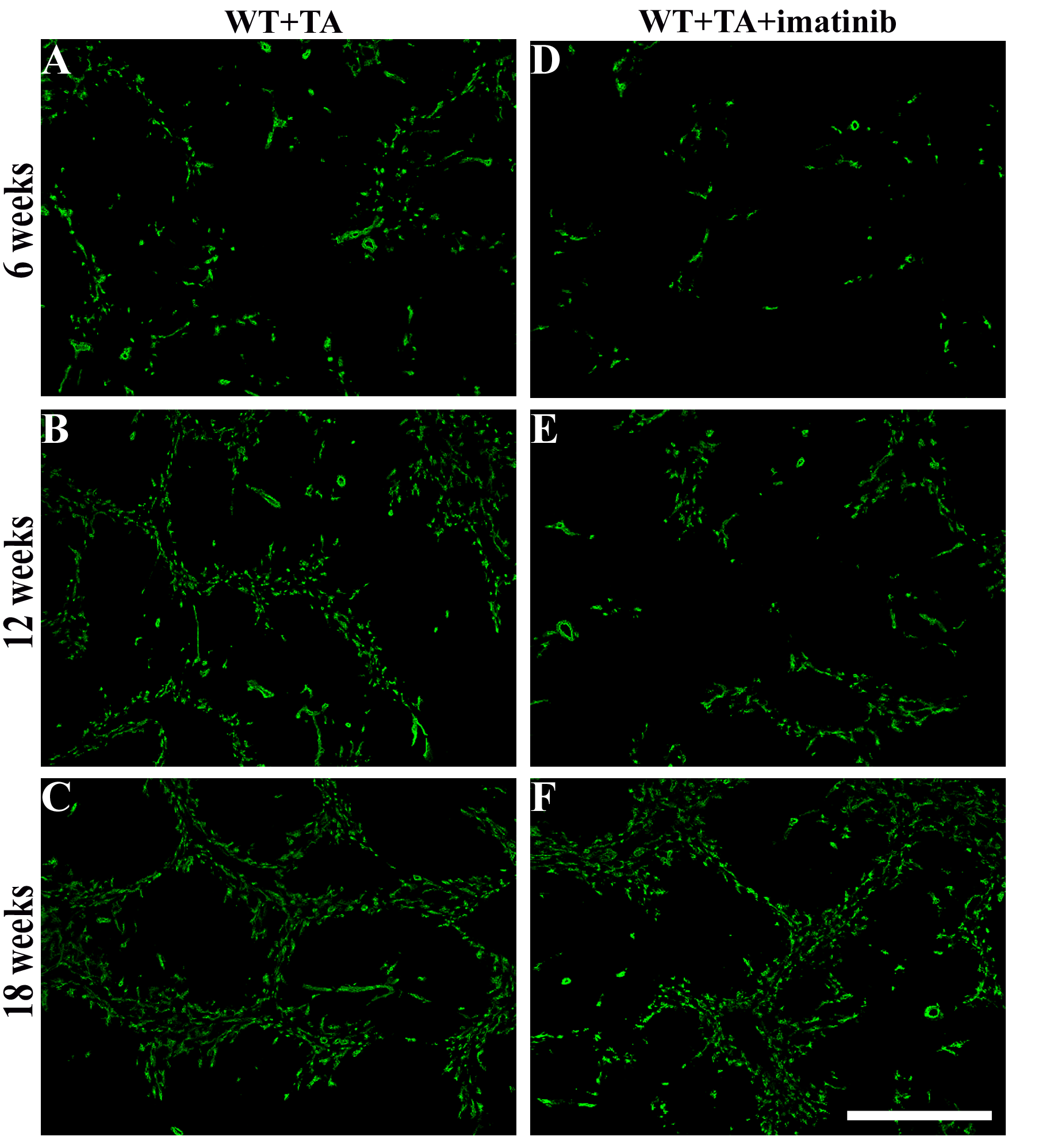

Supplement: S4 Fig — Representative images from sections with CK19 immunofluorescent labeling. Scale bar for S4 Fig.: 200μm. (TIF) [file pone.0176518.s004.tif]

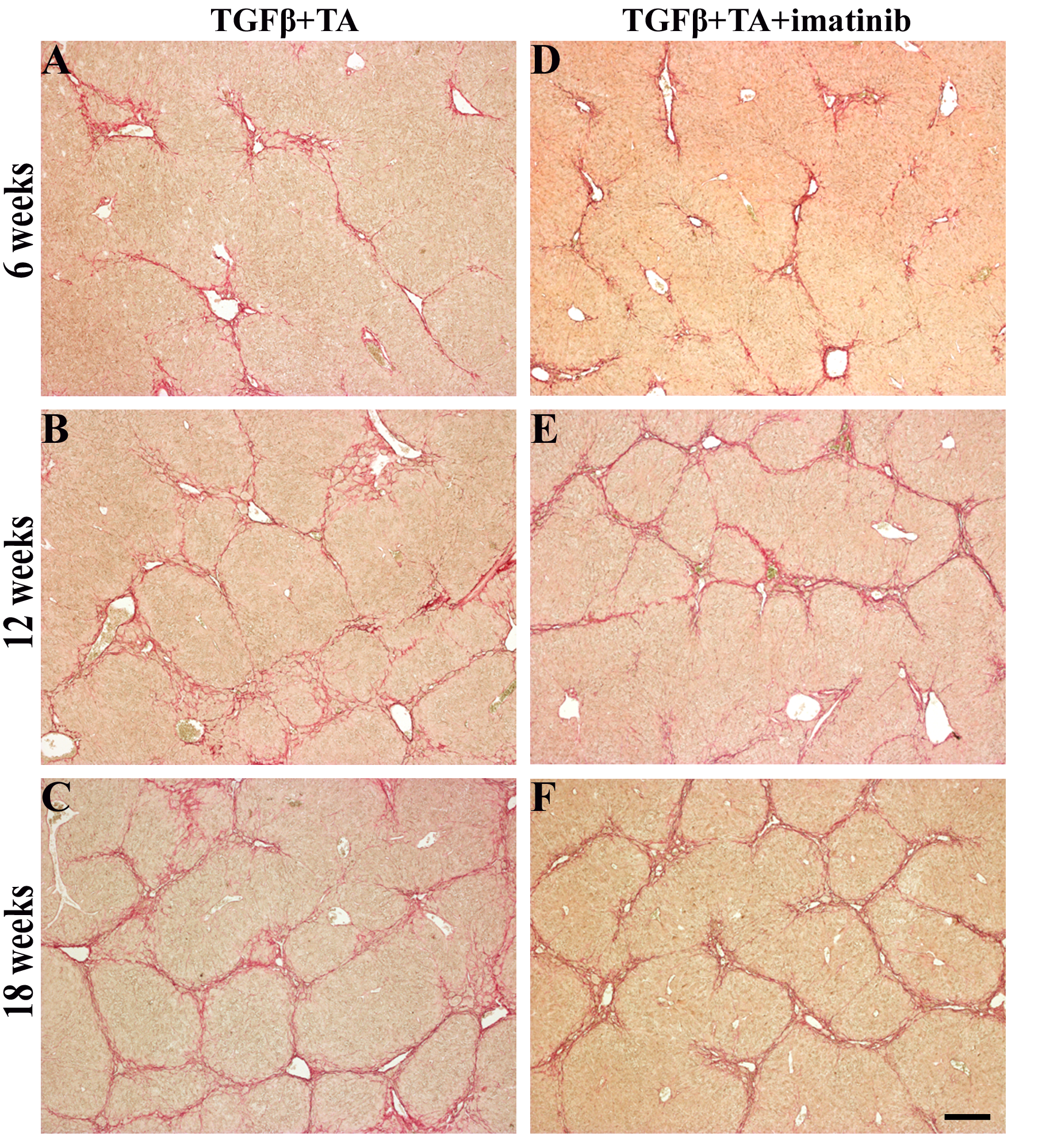

Supplement: S5 Fig — Representative images from Picro Sirius stained sections. Scale bar for S5 Fig.: 200μm. (TIF) [file pone.0176518.s005.tif]

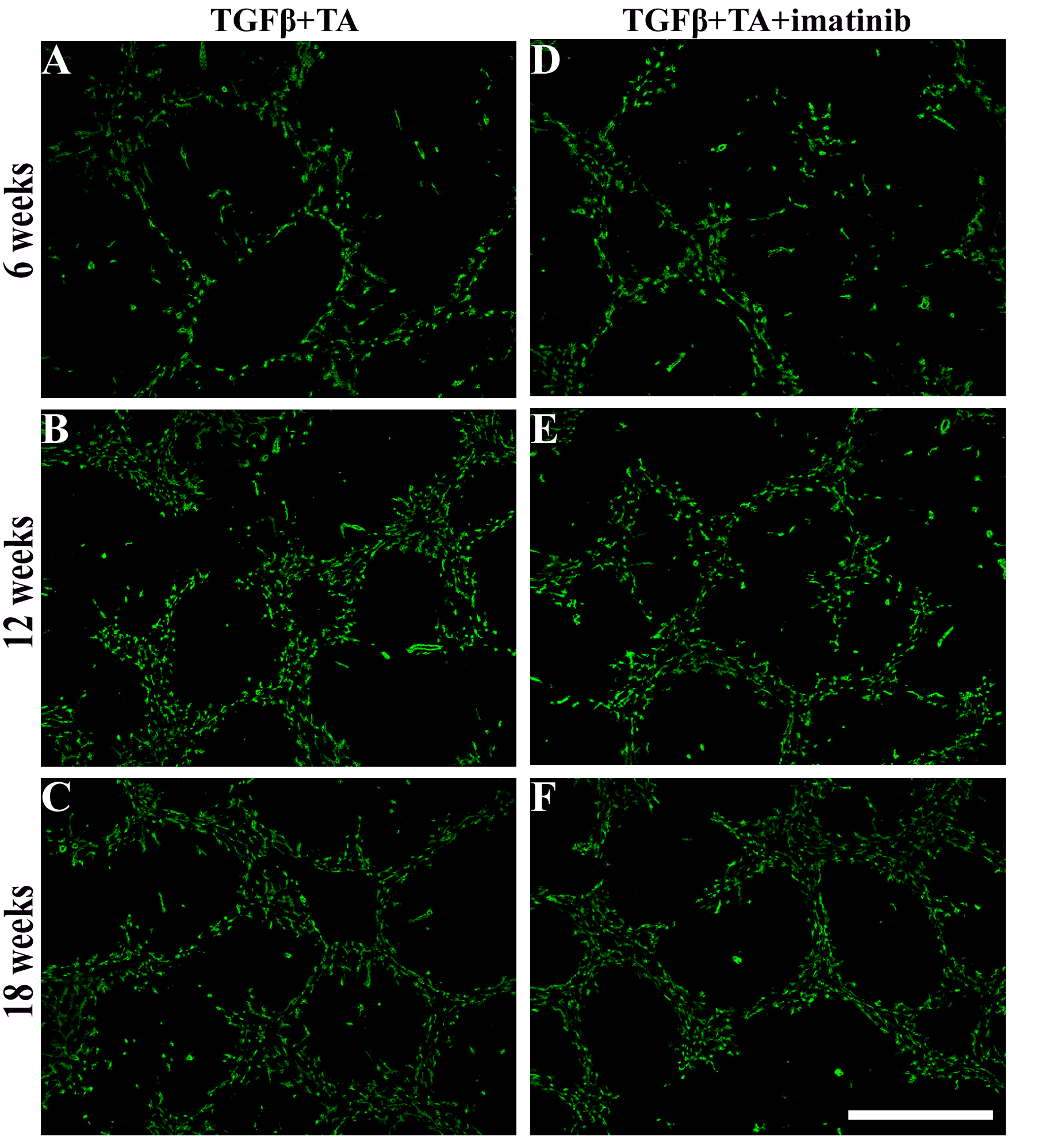

Supplement: S6 Fig — Representative images from sections with CK19 immunofluorescent labeling. Scale bar for S6 Fig.: 200μm. (TIF) [file pone.0176518.s006.tif]

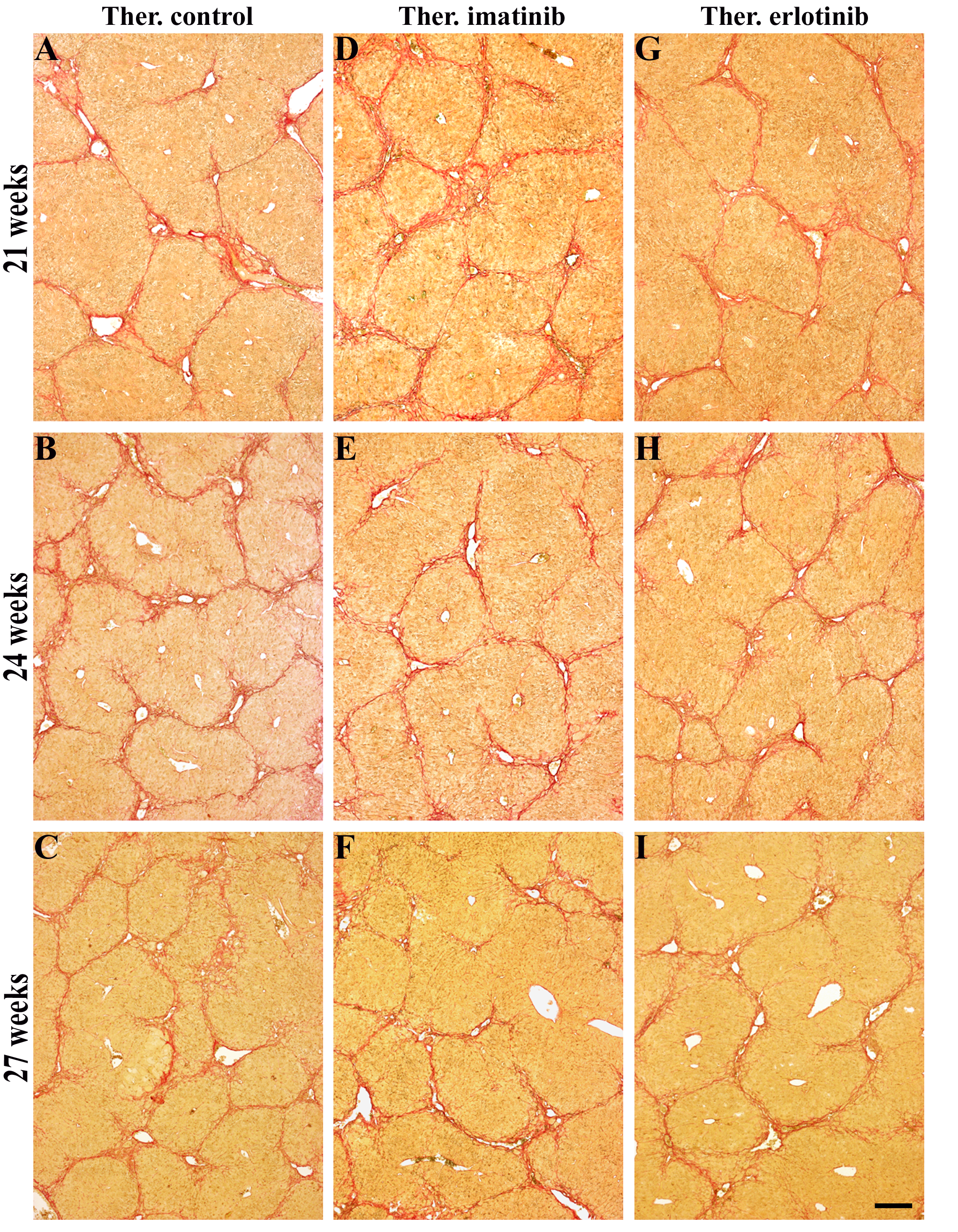

Supplement: S7 Fig — Representative images from sections with Picro Sirius staining. Scale bar for S7 Fig.: 200μm. (TIF) [file pone.0176518.s007.tif]

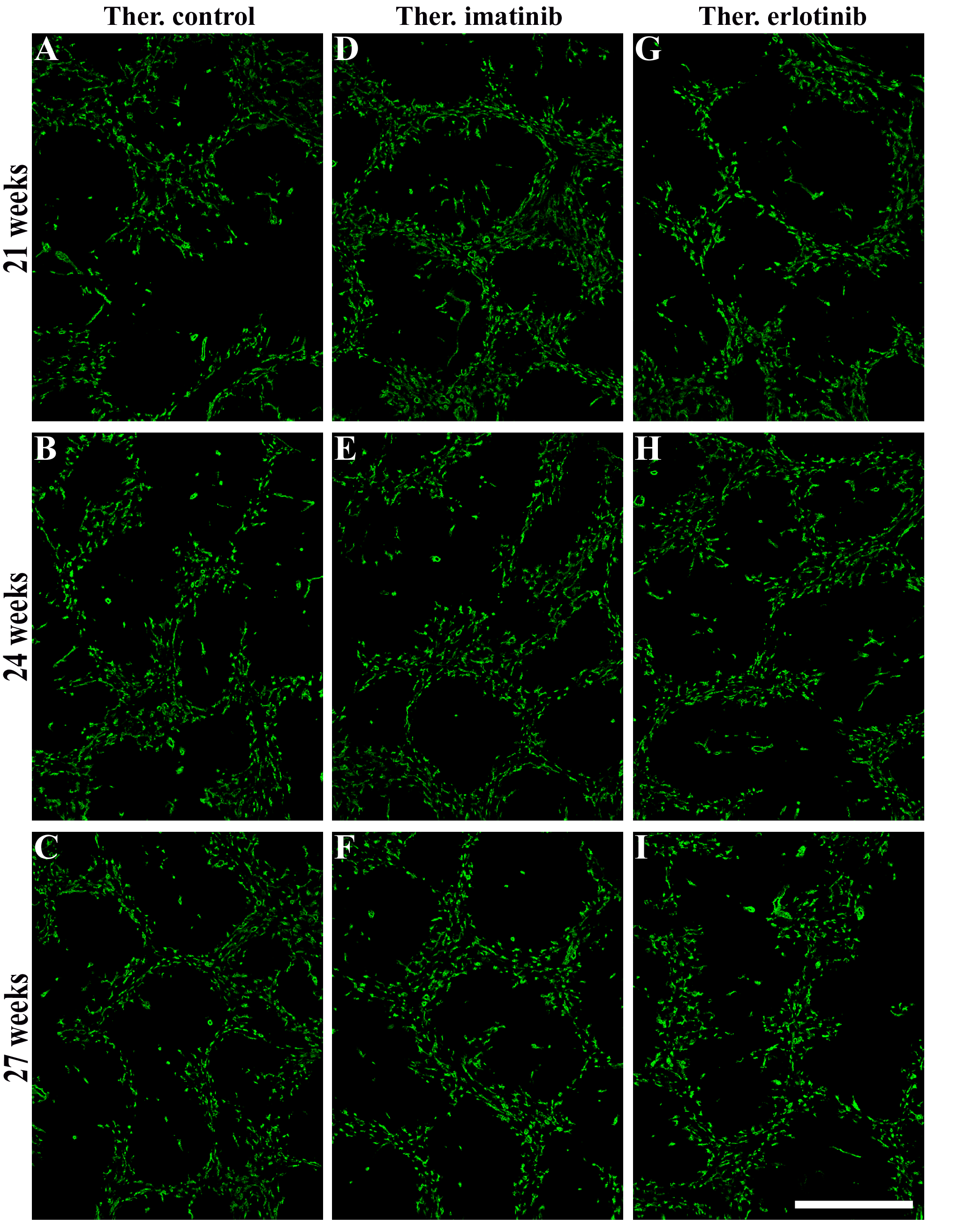

Supplement: S8 Fig — Representative images from sections with CK19 immunofluorescent labeling. Scale bar for S8 Fig.: 200μm. (TIF) [file pone.0176518.s008.tif]

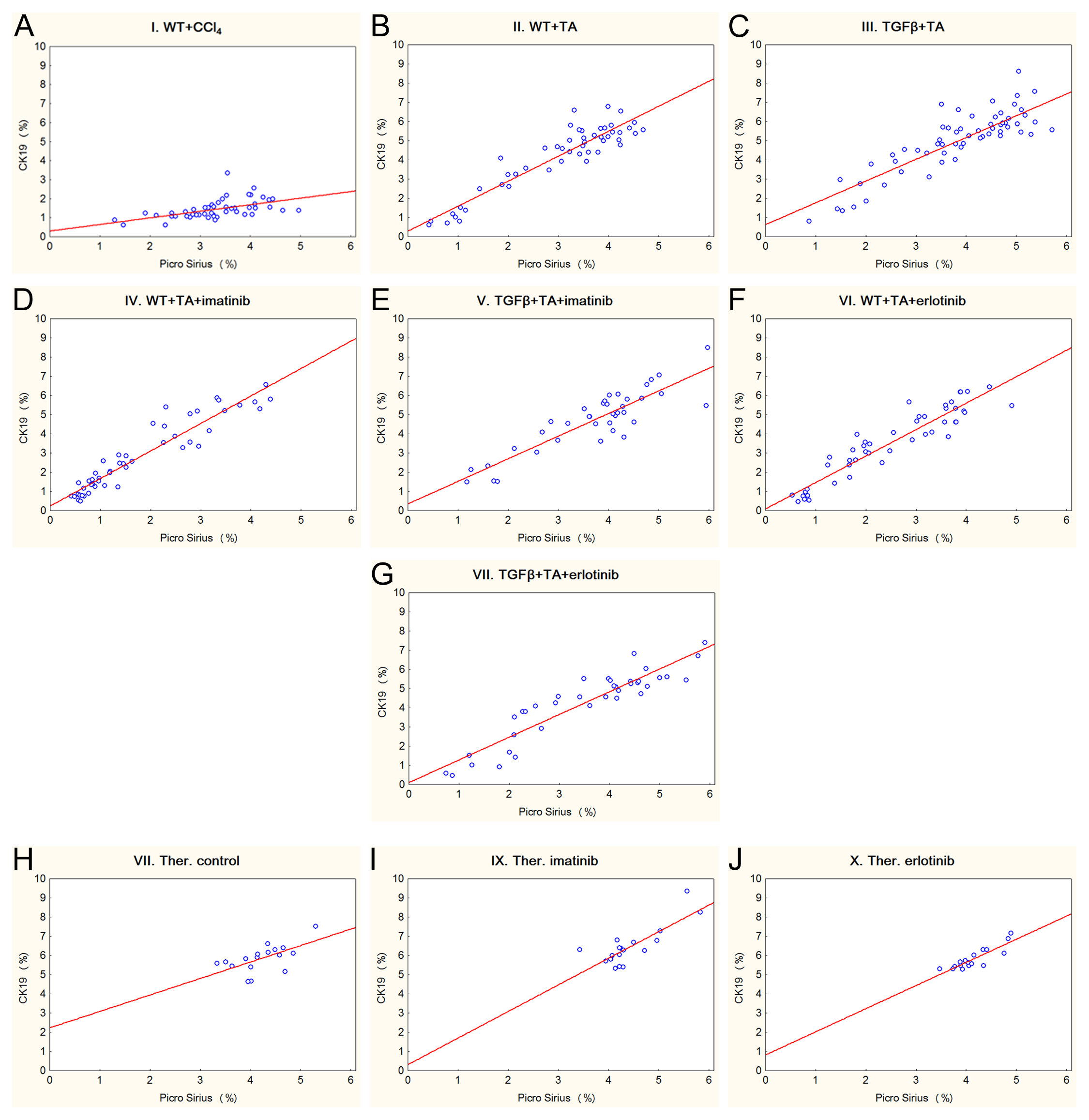

Supplement: S9 Fig — Spearman’s correlation coefficients are shown in Table 1. (TIF) [file pone.0176518.s009.tif]

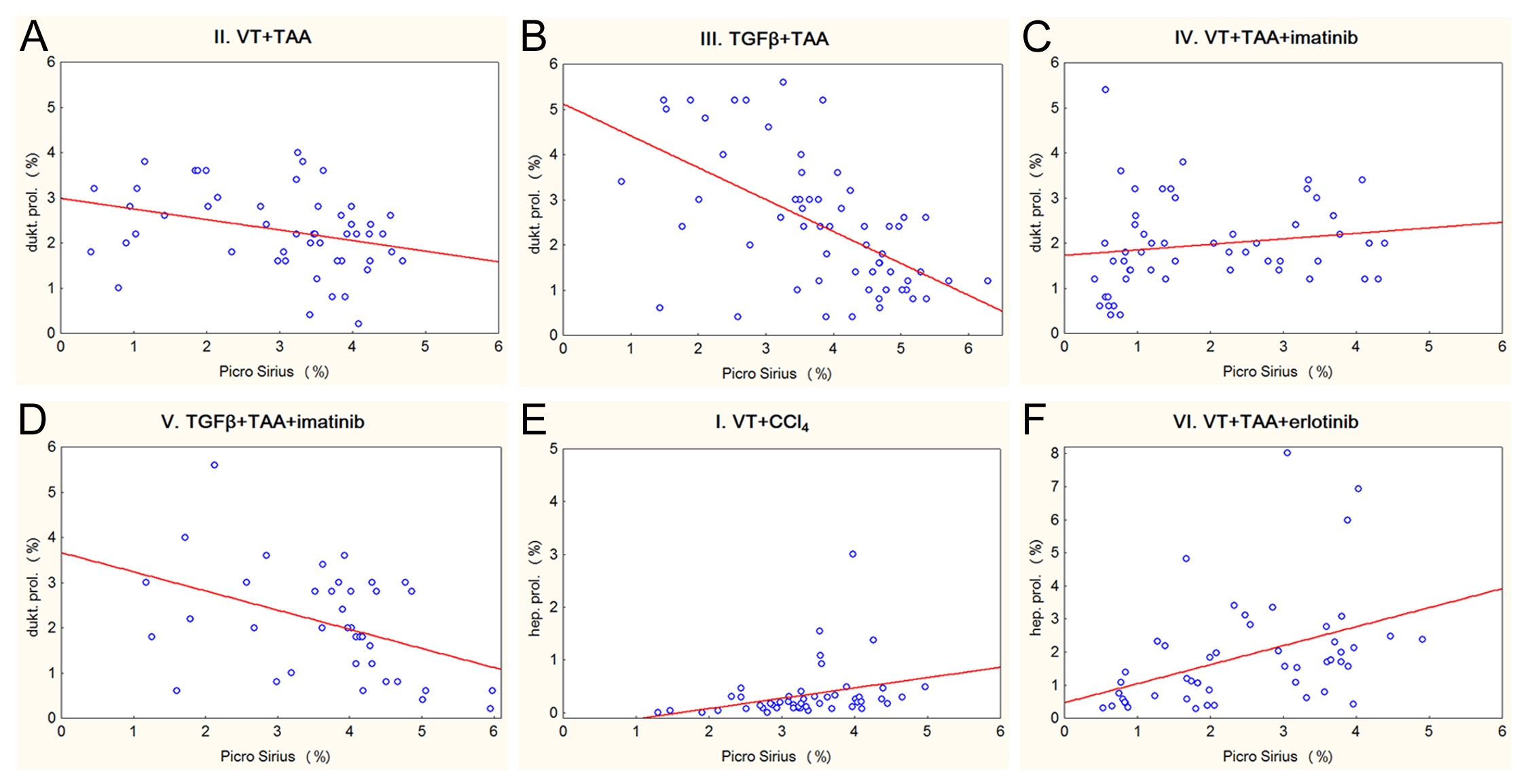

Supplement: S10 Fig — Spearman’s correlation coefficients are shown in Table 1. (TIF) [file pone.0176518.s010.tif]

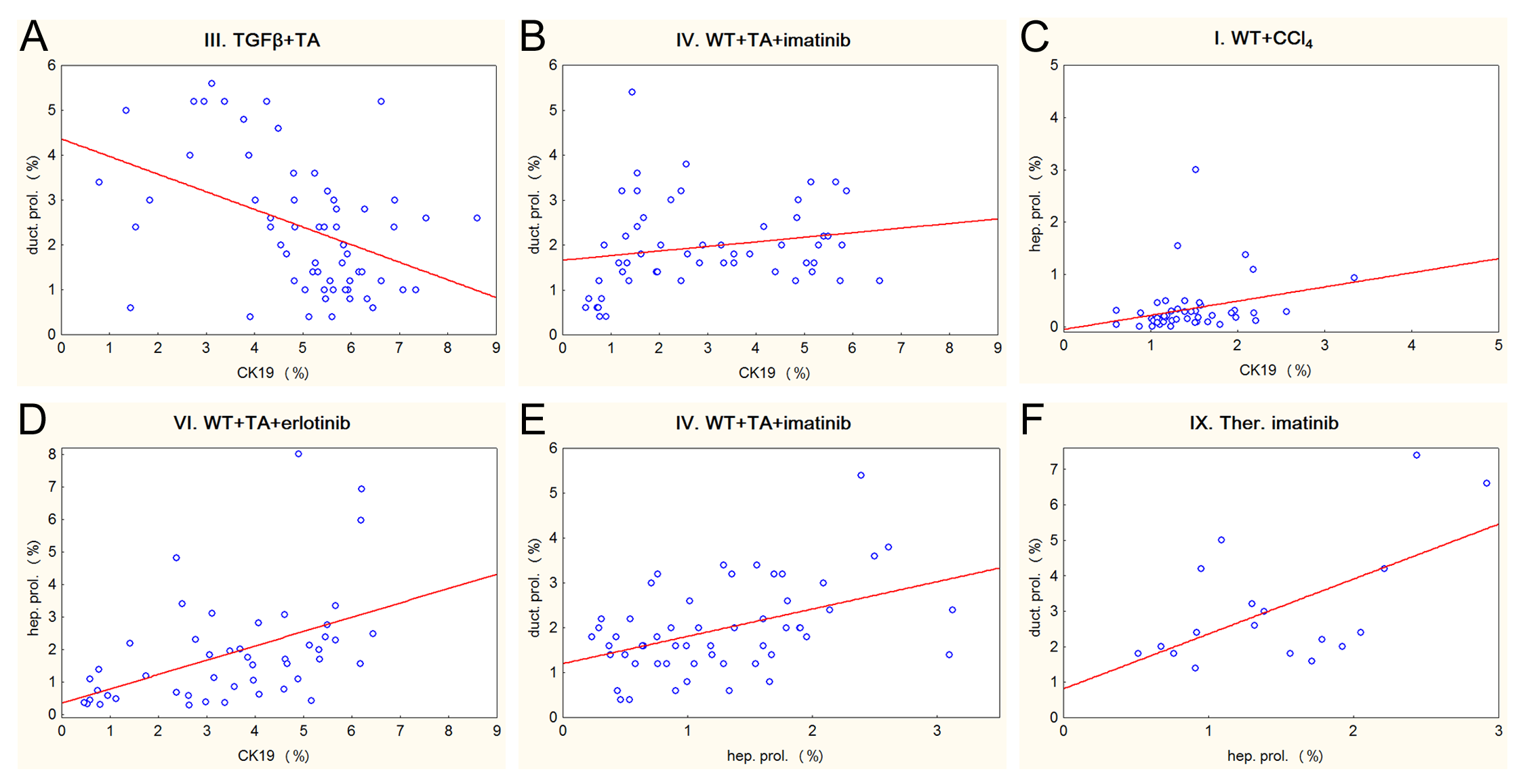

Supplement: S11 Fig — Spearman’s correlation coefficients are shown in Table 1. (TIF) [file pone.0176518.s011.tif]
